# Inter-epidemic seroprevalence of Rift Valley fever virus and associated risk factors in humans in Eastern Rwanda

**DOI:** 10.1371/journal.pntd.0013405

**Published:** 2025-08-22

**Authors:** Isidore Nsengimana, David Kelvin, Evodie Uwibambe, Edson Rwagasore, Claude M. Muvunyi, Gillian Eastwood, Augustino A. Chengula, Christopher J. Kasanga

**Affiliations:** 1 Department of Microbiology, Parasitology and Biotechnology, Sokoine University of Agriculture, Morogoro, Tanzania; 2 SACIDS Africa Centre of Excellence for Infectious Diseases, SACIDS Foundation for One Health, Sokoine University of Agriculture, Morogoro, Tanzania; 3 Rwanda Inspectorate, Competition and Consumer Protection Authority, Kigali, Rwanda; 4 Laboratory of Emerging Infectious Diseases, Department of Microbiology and Immunology, Department of Pediatrics IWK, Faculty of Medicine, The Canadian Centre for Vaccinology, Dalhousie University, Halifax, Canada; 5 Department of Animal Resource Research and Technology Transfer, Rwanda Agriculture and Animal Resources Development Board (RAB), Huye, Rwanda; 6 Rwanda Biomedical Center (RBC), Ministry of Health, Kigali, Rwanda; 7 Department of Entomology, The Global Change Center at VT; and the Center for Emerging Zoonotic & Arthropod-borne Pathogens (CeZAP), Virginia Polytechnic Institute and State University, (Virginia Tech), Blacksburg, Virginia, United States of America; INDEPENDENT RESEARCHER, UNITED STATES OF AMERICA

## Abstract

**Background:**

Rift Valley fever (RVF) is a mosquito-borne zoonosis that causes periodic and explosive epizootics/epidemics in Africa and the Arabian Peninsula. In Rwanda, RVF virus (RVFV) circulation has resulted into two major outbreaks in 2018 and 2022, both of which involving humans. Information on the magnitude of human exposure to RVFV in the country is scarce. This cross-sectional study aimed to investigate the seroprevalence of RVFV and associated risk factors in humans in the Eastern province of Rwanda, 3 years after the end of the 2018 outbreak.

**Methodology:**

A total of 552 outpatients at health facilities in five districts of the Eastern province were randomly sampled and interviewed between December 2021 and February 2022. Exposure to RVFV was examined by detection of anti-RVFV IgG/IgM antibodies in serum samples using a competitive enzyme linked immunosorbent assay (c-ELISA). Bivariate and multivariate logistic regressions were used to assess the association between risk factors and RVFV seropositivity.

**Results:**

The findings revealed an overall seroprevalence of 9.6%. The highest seropositivity, but without significant difference, was observed in Bugesera district (12.9%), followed by Kayonza, (10.8%), Kirehe (8.6%), Rwamagana (7.0%) and Ngoma (6.8%). Odds of seropositivity were significantly higher in people with a history of slaughtering animals (OR 2.26, 95% CI 1.04 - 4.91, p = 0.03), and milking (OR 2.60, 95% CI 1.23 - 5.49, p = 0.012). Sex and age-related differences were not significant.

**Conclusion:**

This study is a first serological survey of RVFV spillover to humans in the country and shows that rural communities in Rwanda have been exposed to RVFV. These findings suggest the Eastern province of Rwanda as a potential hotspot for RVFV circulation, and emphasize the need for a countrywide One Health-based surveillance, prevention, and control strategy to minimize the effects of RVFV in the country.

## Author summary

Rift Valley fever virus (RVFV) is a vector-borne zoonotic pathogen that poses substantial threat to animal and human health in Rwanda. Since its confirmation in the country in 2012, the virus has caused sporadic cases in livestock almost every year, and the first large outbreak occurred in 2018 with suspected human deaths. This study was carried out in rural areas of Eastern Rwanda, 3 years after the 2018 outbreak, to estimate the magnitude of human exposure to RVFV, as well as interview surveys to identify risk factors associated with infection. Understanding the seroprevalence of RVFV and its associated risk factors is essential for developing an effective disease control strategy. The study found an overall seroprevalence of 9.6% with exposure rates ranging between 6.8% and 12.9% across five different study districts. A history of slaughtering animals and milking were risk factors significantly associated with increased risk of RVFV infection in the region.

## 1. Introduction

Rift Valley fever virus (RVFV) is a zoonotic pathogen of One-Health importance, which primarily affects ruminant animals with the potential to cause severe disease in humans. This virus, a phlebovirus classified in the *Phenuiviridae* family, Hareavirales order, class Bunyaviricetes [1] was first described in Kenya in 1930 [2] and is currently endemic in many African countries and the Arabian Peninsula [3]. The virus is transmitted to animals by infected mosquitoes, while humans get infections by mosquito bites or primarily through contact with infected animals, or their fluids or tissues [4]. No horizontal (human-to-human) transmission has been documented [5], but the vertical (mother-to-fetus) transmission associated with miscarriages has been described in pregnant women [6]. In susceptible animals such as cattle, goats, sheep, and camels, the disease is characterized by fever and high rate of abortions in pregnant females and mortality of above 90% in young animals [4]. In humans, RVFV infections are generally asymptomatic or self-limiting febrile illness. However, in a small proportion, severe cases with hemorrhagic syndrome, retinitis, hepatitis and encephalitis can occur [7]. The disease case fatality rate is estimated to be 1–2% [8]. However, because the majority of reported cases involve mainly severe illness requiring medical attention, case fatality rates as high as 47% have been observed, and the disease is often underreported [7]. Presently, there is no specific treatment for RVF and only a few vaccines are commercially available for veterinary use only in endemic regions. Fully licensed vaccines are still missing for use in humans [9–11] and animal vaccination remains the available strategy to prevent or control the outbreak and minimize spillover to humans [5].

When RVFV incurs in an area, it is most likely maintained in the environment [12]. The silent circulation, also termed endemic/enzootic cycle, is the maintenance mechanism whereby the virus circulates below a detectable level between mosquitoes and their vertebrate hosts [13]. This mechanism, which may involve humans, has been suggested to predominate in areas with a permanent mosquito presence; thus, the prolonged dormancy of the virus in mosquito eggs, as previously reported, may not be necessary for its maintenance in these regions [14]. While the regular monitoring of seroconversion in these areas can provide information on the extent of virus circulation and risk of exposure in susceptible hosts [15], the lack of adequate surveillance systems and laboratory capacity often becomes a challenge in Africa [16]. Viral latency in dry-resistant mosquito eggs is another proposed mechanism for maintaining the virus, suggested to occur in some *Aedes* spp. mosquito species capable of transmitting the virus vertically [12,17,18]. Rift Valley fever virus - competent mosquito vectors have been identified in different genera, including *Aedes*, *Culex, Anopheles,* and *Mansonia* [19,20]. The presence of RVFV - competent mosquitoes in all continents coupled with the virus’s demonstrated capacity to cross borders, make RVFV a pathogen with the potential to spread globally [21–26].

Outbreaks of RVFV have been occurring in Africa with intervals ranging from 3 to 15 years [27,28], and are mainly triggered by heavy and prolonged rainfall [29]. During an epizootic/epidemic, RVFV is excessively amplified by susceptible animal hosts with a high probability of spilling over to humans. Historically, severe RVFV outbreaks that caused the most significant damage to public health occurred in Egypt in 1977/8 (over 18,000 reported cases with 598 deaths) [30], Saudi Arabia and Yemen in 2000 (1,603 cases with 208 deaths), Kenya, Tanzania and Somalia in 2006/07 (1,062 cases with 394 deaths) and Sudan in 2007 (738 cases with 230 deaths) [31]. People involved in slaughtering or caring for sick or birthing animals have been reported to be at high risk of contracting RVFV [32,33]. In humans, the RVFV incubation period is 2–6 days, and the virus remains detectable in blood up to 4–5 days after the onset of symptoms [34]. Specific anti-RVFV immunoglobulin M (IgM) antibodies, biomarkers of a recent infection, appear after 5–6 days of infection and last for a few months, while anti-RVFV immunoglobulin G (IgG) antibodies, which indicate past infection, appear after 1–2 weeks and last for many years [35]. Generally, RVFV infection is cleared by the human body within 6 days of symptoms or it evolves into a severe form with a likelihood of death occurring between 6–10 days [34]. In Africa, RVF is often misdiagnosed as malaria, and coinfections of these two vector-borne diseases, which usually share an overlapping geographical distribution, have been observed, though they remain underreported [36].

Despite Rwanda being located in the East African region, a known endemic area of RVF, little information is available on the epidemiology of this disease. The presence of RVF in Rwanda was confirmed for the first time in 2012 [37] and the country experienced the first large epizootic in 2018 that caused hundreds of animal cases with two suspected human deaths [38]. A second nationwide RVF outbreak occurred in 2022 when 173 human cases and 27 deaths were reported [39,40]. Because the majority of RVFV cases are asymptomatic [7], the magnitude of human exposure remains difficult to establish without studies such as seroprevalence assessment. Knowledge of the seroprevalence in a population is critical to the evaluation of the virus circulation, and the assessment of the risk of outbreaks, both of which are key to guiding the prevention and control interventions [41,42]. This study therefore aimed to determine the inter-epidemic seroprevalence and potential risk factors of RVFV in rural areas of the Eastern province of Rwanda, 3 years after the end of the 2018 RVF epizootic.

## 2. Methodology

### 2.1. Ethics statement

This research was conducted in collaboration with Rwanda Biomedical Center, an implementing Agency of the Rwanda Ministry of Health, collaboration note No. 3062/RBC/2021. In addition, research permit and ethical approvals were obtained from Ethics Committee of Sokoine University of Agriculture (Morogoro, Tanzania), reference, RPGS/R/ETHICS/05/08/2021, Rwanda National Health Research Committee, reference NHRC/2021/PROT/042, Rwanda National Council for Science and Technology (NCST), research permit No. NCST/482/257/2021 and Rwanda National Ethics Committee (RNEC), authorization No. 836/RNEC/2021.

### 2.2. Study area and design

**Fig 1:**
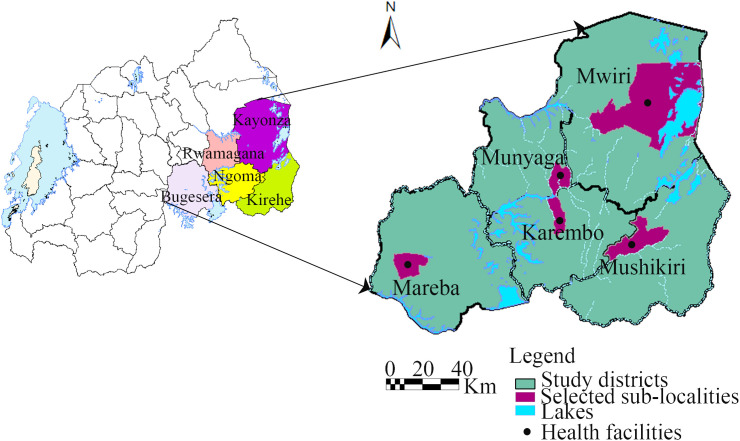
Map of Rwanda showing the five districts of the study area and selected sub-localities. Black dot indicates location of health facilities used for sample collection. The map was drawn using the QGIS version 3.24.1 freely available at: https://www.qgis.org/en/site/, accessed on 25 November 2024.

This health facility–based cross-sectional study was conducted from December 2021 to February 2022 in five districts (Bugesera, Rwamagana, Kayonza, Ngoma, and Kirehe) of the Eastern province of Rwanda (Fig 1), which was the epicenter and the most affected region (except Bugesera and Nyagatare districts) during the 2018 RVFV epizootic [38]. The Eastern province, which shares borders with Uganda, Tanzania, and Burundi, is a dry and hot lowland agro-pastoral area rich in rivers and marshlands [43]. Irrigated rice and banana plantations are among the predominant crops [44]. The average temperature in the area ranges between 20–22°C and the mean annual rainfall varies between 700–1100 mm with two rainy seasons, from mid-September to December, and from March to May [43]. In each study district, one administrative sub-locality (named sector) with a history of RVF outbreak where applicable, was selected and the health facility (locally known as Health Center) operating in that sub-locality was used for collecting blood samples among outpatients attending that health facility.

### 2.3. Participant recruitment and blood sample collection

A total of 552 participants were recruited for this study. The number of human subjects recruited in each study sub-locality was determined based on their probability proportional to the updated total size of human population in that sub-locality. Outpatients ≥ 7 years of age and residents of the selected sub-locality who presented at the health center for medical services were invited to participate in the study. A first-come, first recruited approach was used to select participants attending on a given sampling day; in the case of many individuals from the same family, a maximum of two family members were included in the study. Individuals ≥ 18 years were requested to voluntarily provide their written consent to participate in the study, while parents consented on behalf of their children under 18 years. For children between 10–18 years, both parent consent and child’s assent were obtained together, in written forms. For every recruited participant, blood was collected by a phlebotomist of the health facility in accordance with existing standard procedure. At the end of each collection day, blood collected using a plain vacutainer tube was centrifuged and the resulting serum stored at -20^o^ C pending laboratory analysis.

### 2.4. Assessment of risk factors

A structured questionnaire was used to collect data from individuals aged 18 years and above. In case of under-18-year children, questionnaires were completed by their parents. The information collected related to demographic characteristics, animal and non-animal exposure risk factors, referring to a 3-year recall period prior to the interview date. Animal exposure risk factors assessed included direct contact with animals or fluids, ownership of livestock, history of handling dead animals, slaughtering, handling aborted material, assisting in animal birth, and milking. Non-animal exposure risk factors such as sex, age, level of education, occupation, irregular use of insecticide treated nets (ITN), and geographical location were also assessed.

### 2.5. Determination of seroprevalence

The presence of the anti-RVFV antibodies in the collected serum samples was examined using the commercial kit, ID Screen® Rift Valley Fever Competition Multi-species ELISA (Innovative Diagnostics, Grabels, France), according to the manufacturer’s instructions. This kit detects, at the same time, both IgG and IgM antibodies directed against the nucleoprotein (NP) of the virus. There was no test done to separate IgM-positive subjects (recent infections) from IgG-positive individuals (past infections). The preliminary evaluation of the test found a diagnostic sensitivity of 100% with a 95% confidence interval (CI) of 91.2 - 100% and a specificity of 100%, CI 95%: 99.6 – 100%, [23].

### 2.6. Data analysis

The questionnaire data were entered into Microsoft Excel and matched with ELISA results. A binary variable indicating RVFV status was generated, with a value of 1 if an ELISA positive result was obtained and 0 in case of a negative result. The prepared Excel file was imported into STATA 13.1 (StataCorp, College Station, TX, USA) for analysis. RVFV seroprevalence was determined as the proportion of individuals in the tested population that had antibodies against RVFV. The significance of the association between each risk factor and RVFV seropositivity was initially assessed using bivariate analysis based on the Pearson Chi-square test. A p-value of < 0.20 was set as a criterion for selecting variables for the downstream analysis. All independent variables retained after bivariate analysis were analyzed for adjusted association using multivariable logistic regression. A p-value of < 0.05 was considered statistically significant.

## 3. Results

### 3.1. Sociodemographic characteristics of participants

A total of 552 people participated in the study. Participants were recruited from five districts of the Eastern province namely Rwamagana (86 participants, 15.6%), Kayonza (130 participants, 23.6%), Ngoma (73 participants, 13.2%), Kirehe (139 participants, 25.2%) and Bugesera (124 participants, 22.5%), as summarized with demographic breakdown in Table 1. Participants were aged between 7 and 83 years, with a mean age of 33.2 years (standard deviation: ± 13.3). The majority of participants were females (60%, n = 331), aged between 20 and 39 years (60.9%, n = 336), had attended primary school (81.3%, n = 449), were engaged in farming as their primary occupation (89.5%, n = 494), or owned livestock at home (83.2%, n = 459).

**Table 1:** Participant demographic characteristics.

Variable	Level	Total (n = 552)	%
Sex	Female	331	60.0
	Male	221	40.0
Age	Mean = 33.2 (±13.3) min. = 7, max = 83		
	< 20	66	12.0
	20 - 39	336	60.9
	≥ 40	150	27.2
Education level	None	59	10.7
	Primary	449	81.3
	Secondary	42	7.6
	Tertiary	2	0.4
Occupation	Farmer	494	89.5
	Off-farm private	4	0.7
	Public servant	2	0.4
	Student	52	9.4
Family owns livestock	Yes	459	83.2
	No	93	16.8

### 3.2. Serology results

Of all individuals tested, 9.6% (53/552) were seropositive against RVFV with a 95% CI of 7.3 - 12.4. The highest, but not significant (χ2 = 3.23, P = 0.52), seroprevalence was observed in Bugesera district, 16/124 (12.9%; CI: 7.0 – 18.8), followed by Kayonza, 14/130 (10.8%; CI: 6.0 - 17.4), Kirehe, 12/139 (8.6%; CI: 2.6 - 14.5), Rwamagana, 6/86 (7.0%; CI: 2.6 - 14.5) and Ngoma, 5/73 (6.8%; CI: 2.2 – 15.2). Male participants showed a higher, but not statistically significant (χ^2 ^= 5.26, P = 0.22), seropositivity rate (29/221, 13.2%) compared to females (24/331, 7.2%). Among occupational categories, farmers and students exhibited similar seropositivity rates (48/494 (9.7%) and 5/52 (9.6%), respectively). Additionally, comparable seropositivity rates were noted among individuals under 20 years (8/66, 12.1%) and those above 40 years (18/150, 12%). Bivariate analysis revealed significant correlations between RVFV seropositivity and certain activities: slaughtering animals (χ^2 ^= 16.85; p < 0.001), assisting in livestock birthing (χ^2 ^= 4.98; p = 0.026) and milking (χ^2 ^= 13.52; p < 0.001), (Table 2).

**Table 2:** Bivariate analysis of potential risk factors of RVFV seropositivity.

Variable	Level	Total	No. positive	% (95% CI) seropositivity	Bivariate analysis X^2^ (P value)
District	Bugesera	124	16	12.9 (7 – 18.8)	3.23 (0.52)
	Kirehe	139	12	8.6 (4.5 – 14.6)	
	Rwamagana	86	6	7 (2.6 - 14.5)	
	Ngoma	73	5	6.8 (2.2 – 15.2)	
	Kayonza	130	14	10.8 (6 - 17.4)	
Sex	Male	221	29	13.1 (8.8 - 18.3)	5.26 (0.22)
	Female	331	24	7.2 (4.7 - 10.6)	
Occupation	Farmer	494	48	9.7 (7.2 - 12.7)	0.75 (0.68)
	Students	52	5	9.6 (3.2 - 21)	
	Others	6	0	0 (0 - 4)	
Education	None	59	9	15.2 (7.2 - 27)	2.78 (0.25)
	Primary	449	39	8.7 (6.2 - 11.7)	
	2nd&3tiary	44	5	11.4 (3.8 - 24.6)	
Age	under 20	66	8	12.1 (5.4 - 22.3)	2.42 (0.29)
	20-39	336	27	8 (5.4 - 11.5)	
	Above 40	150	18	12 (7.2 - 18.3)	
ITN irregular use	Yes	202	25	12.4 (8.2 - 17.8)	2.82 (0.09)
	No	350	28	8 (5.4 - 11.3)	
Family owns livestock	Yes	459	43	9.4 (6.9 - 12.4)	0.17 (0.67)
	No	93	10	10.7 (5.3 - 18.9)	
Handled dead animal	Yes	72	9	12.5 (5.9 - 22.4)	0.80 (0.37)
	No	480	44	9.2 (6.7 - 12)	
Sheltering animals at home	Yes	414	40	9.6 (7 - 13)	0.00 (0.93)
	No	138	13	9.3 (5 - 15.4)	
Slaughtered an animal	Yes	79	17	21.5 (12.9 - 31.8)	16.85 (< 0.001)
	No	426	31	7.3 (5.8 - 11.1)	
Handled meat for cooking	Yes	339	34	10 (7 - 13.7)	0.18 (0.66)
	No	213	19	8.9 (5.4 - 13.5)	
Worked in a butcher shop	Yes	21	5	23.8 (8.2 - 47.2)	5.14 (0.07)
	No	482	43	8.9 (6.6 - 11.8)	
Milked a cow	Yes	97	19	19.6 (12.2 - 28.9)	13.52 (< 0.001)
	No	455	34	7.5 (5.2 – 10.3)	
Assisted in livestock birthing	Yes	121	18	14.9 (9 - 22.5)	4.98 (0.026)
	No	431	35	8.1(5.7 - 11.1)	
Handled aborted material	Yes	42	6	14.3 (5.4 - 28.5)	1.14 (0.28)
	No	510	47	9.2 (6.8 - 12)	

CI: confidence interval; OR: odds ratio; ITN: Insecticide-treated nets

### 3.3. The association of potential risk factors to the RVFV seropositivity

In the multivariable logistic regression analysis, slaughtering animals (OR: 2.26, 95% CI 1.04 - 4.91, p = 0.03) and milking (OR: 2.60, 95% CI 1.23 - 5.49, p = 0.012) were significantly associated with an increased risk of RVFV seropositivity. On the other hand, assisting in livestock birthing, irregular use of insecticide-treated nets, and working in a butcher shop, were not found to be significantly associated with RVFV seropositivity in the multivariable analysis (Table 3).

**Table 3:** Multivariable analysis of potential risk factors of RVFV seropositivity.

		Multivariable analysis results		
Variable	Level	OR	95% CI	P value
ITN irregular use	Yes	0.55	0.29 - 1.02	0.06
	No			
Slaughtered an animal	Yes	2.26	1.04 - 4.91	0.03
	No			
Worked in a butcher shop	Yes	1.62	0.48 - 5.47	0.43
	No			
Milked a cow	Yes	2.60	1.23 - 5.49	0.012
	No			
Assisted in livestock birthing	Yes	0.95	0.41 - 2.17	0.90
	No			

OR: odds ratio; CI: confidence interval; ITN: Insecticide-treated nets

## 4. Discussion

Rift Valley fever virus has been serologically and molecularly confirmed in Rwanda’s livestock since 2012 [37,45], with sporadic cases reported almost annually. Despite two major outbreaks that have occurred in the country [38,39], data on the extent of human exposure to RVFV and the associated risk factors have been scarce. Here, our baseline study, conducted 3 years after the end of the 2018 RVFV outbreak, indicated that 9.6% of individuals living in the rural area of the Eastern province were seropositive to RVFV. Seropositivity rates obtained in some study districts were comparable to seroprevalence observed in 2016 in Kabale district, Uganda (13%), which borders Rwanda. During this localized outbreak, two acute human cases were confirmed in Uganda for the first time in 48 years of inter-epidemic period [46]. A similar seroprevalence was reported in Northeastern Kenya 2 years after the 2006 major RVF epizootic there [47] and in the Kilimanjaro region of Northeastern Tanzania in 2020 [48]. Our results are also comparable to the 11.7% inter-epidemic seroprevalence reported in 2012 in a flood-prone Kilombero River Valley of Tanzania [49]. However, they are higher than the 1.4% seropositivity observed in 2020 in Sengerema, a traditionally pastoral district of Tanzania, after 13 years of an inter-epidemic period [50]. This discrepancy in seropositivity rates could be attributed to differences in local ecological conditions, animal or human population densities, history of previous outbreaks, sampling strategies or diagnostic methods used.

Although no livestock or human cases were officially reported in Bugesera district during the 2018 Rwanda RVF outbreak [38], the analysis showed that RVFV human exposure has occurred in that region, and there was no significant difference detected in our study in the distribution of RVFV human seropositivity across the five districts under study. Historically, Bugesera district has been the area where livestock abortions suspicious of RVFV were first observed and later confirmed in Rwanda in 2012 [37]. The absence of the 2018 outbreak in Bugesera district was attributed to higher vaccination coverage compared to other districts in the same area [38]. However, the lack of a significant difference in the spatial distribution of human seropositivity in our data, suggested that a similar risk of human exposure to RVFV exists in all the study districts. A study conducted in 2012 showed that RVFV was steadily circulating in cattle in many of these districts [45] and several RVFV sporadic cases were later recorded in the Eastern province during periods without a major outbreak [51,52].

Our study further indicates that individuals with a history of animal-exposure practices, such as slaughtering animals and milking, have an increased risk of RVFV exposure and resulting seropositivity. These risk factors have been reported in previous studies [32]. Elsewhere, practices such as animal slaughtering have been described as predictors likely associated with the highest risk of RVFV infection, particularly during periods of higher virus activity [3]. Individuals engaged in slaughtering livestock are at risk of infection through wounds on their hands or by inhaling aerosols produced during slaughtering activities [50,53]. Previous studies also observed that working in a butcher shop may be associated with an increased likelihood of RVFV seropositivity [54]. However, we did not find this association in our study after adjusting for other factors, likely because only a small number of participants (21; 3.8%) reported working in butcher shops, which are rare in rural parts of Rwanda. Further studies are needed to explore this aspect, particularly in urban settings. Nonetheless, an association between milking animals and increased odds of seropositivity was identified. Similar findings have been reported in Tanzania [49] and in Kenya [54]. In Rwanda and many African countries, slaughtering and milking are more commonly performed by males than females. While there is no general consensus on the increased risk of RVFV seropositivity in males compared to females [3], we noted a higher seropositivity in males compared to females, although the difference was not statistically significant.

No significant difference in human seropositivity was observed between formally and non-formally educated individuals, farmers and students, and between the three age groups. This lack of significant difference is unsurprising given that, in rural areas of Rwanda where a big proportion of households keep animals, it is common for all family members, regardless of their education, occupation or age to participate in animal care. Additionally, the shared risk of mosquito bites in farming households [55] further contributes to uniform exposure. With regard to age, it is often a general characteristic of endemicity that higher seropositivity rates are observed in older individuals compared to younger ones due to prolonged exposure over lifetime [3,48]. Contrary to this, our findings, being consistent with studies conducted in Kenya [47], showed no significant difference between age groups. This aligns with the context in Rwanda, a country where the majority of the population is young and resides in rural areas participating in daily animal husbandry activities [56]. The results also support a prior observation that RVF endemicity in Rwanda is recent but more active [57], placing the rural population at risk of infections. Since RVFV seropositivity may vary by locality and study methodology [58], only limited conclusions can be drawn when comparing our serological results to findings from other neighboring countries. However, the observation of seropositivity rates comparable to those reported in countries known to have a long history of endemicity [46,47,49], suggest a considerable risk in Rwanda. Although not all RVFV cases result in human fatalities [5], the disease’s burden can be significant in ecosystems with constant exposure risks. Therefore, efficient nationwide One Health surveillance is paramount to track transmission and minimize the impact of RVFV in the country [41].

Our study has some limitations. The sampling sites used in this health facility–based cross-sectional study were purposively selected, focusing on areas that reported more cases during the 2018 outbreak, except Bugesera district where no outbreak was reported. Thus, caution is required when extrapolating our results to a national scale. Additionally, the study focused on IgG/IgM antibody detection at same time without separating past (IgG positive) from recent (IgM positive) infections, thus lacking information on the exact period of exposure. This limitation hampers the ability to determine the extent of active infections in humans. Longitudinal studies separating positivity to each of the two antibodies are recommended for a more comprehensive understanding of RVFV infection dynamics.

## 5. Conclusion

This study is a first sero-epidemiological survey of RVFV in humans in Rwanda. The findings suggest that a considerable number of people in rural areas have been exposed to RVFV, and individuals with a history of animal-exposure practices, such as slaughtering, and milking, are at higher risk of infections. The data suggest the Eastern province of Rwanda as a potential hotspot for RVFV circulation, and underscore the need for efficient One Health-based surveillance, prevention, and control strategies to combat this zoonotic disease.

## Supporting information

S1 Dataset Data used for analysis. (CSV)
